# Giant-cell tumour of proximal radius in a 50-year-old female with wrist drop: a rare case report

**DOI:** 10.1007/s11751-017-0281-y

**Published:** 2017-03-31

**Authors:** Anshul Dahuja, Rashmeet Kaur, Shiraz Bhatty, Simmi Garg, Kapil Bansal, Mandeep Singh

**Affiliations:** GGS Medical College, 242 medical campus, Faridkot, Punjab India

**Keywords:** Arthrodesis, Giant-cell tumour, Proximal radius, Sub-chondral bone, TENS (titanium elastic nail system)

## Abstract

Giant-cell tumour is a locally aggressive tumour of long bones of epiphyseal region commonly occurring in adults aged 20–40 years. Most common location is distal femur, proximal tibia, and distal radius. Different treatment options being used are curettage with bone graft or bone cement, resection with arthrodesis, reconstruction, radiation, and chemotherapy. We are reporting a case of giant-cell tumour of right proximal radius in a 50-year-old female with posterior interosseous nerve palsy. It is very rare, and only four cases have been reported in the literature. It was treated by wide margin resection with fibular grafting, titanium elastic nail system along with cancellous bone graft reconstruction.

## Introduction

Giant-cell tumour is a locally aggressive tumour of long bones in epiphyseal region [1]. It occurs in 20–40-year age group with slight female predominance, though rarely found in other age groups [2]. Most common locations of this tumour are distal femur, proximal tibia, distal radius, and spine [3]. It is a solitary, benign, and locally aggressive tumour, less than 5% are malignant. Usually, patient presents with progressive pain, mass and pathological fracture. On radiographs, the lesions are lytic, eccentric in the epiphyses of long bones, and usually abut the sub-chondral bone, though sometimes in metaphysis of skeletally immature patients [1]. Various treatment modalities are available depending upon the stage of the tumour. For active lesion, simple curettage or extended curettage with adjuvants, bone graft, or bone cement is preferred [4]. For aggressive lesion, primary resection of tumour with or without reconstruction of site with fibular graft or endoprosthesis is used [5]. Inoperable and metastatic lesions are treated by radiation or chemotherapy. Post-operatively, patient should be followed up regularly for recurrence. We hereby report a case of 50-year-old female having a giant-cell tumour in proximal radius along with wrist drop. This is unusual regarding both age and location of GCT.

### Case report

**Fig. 1:**
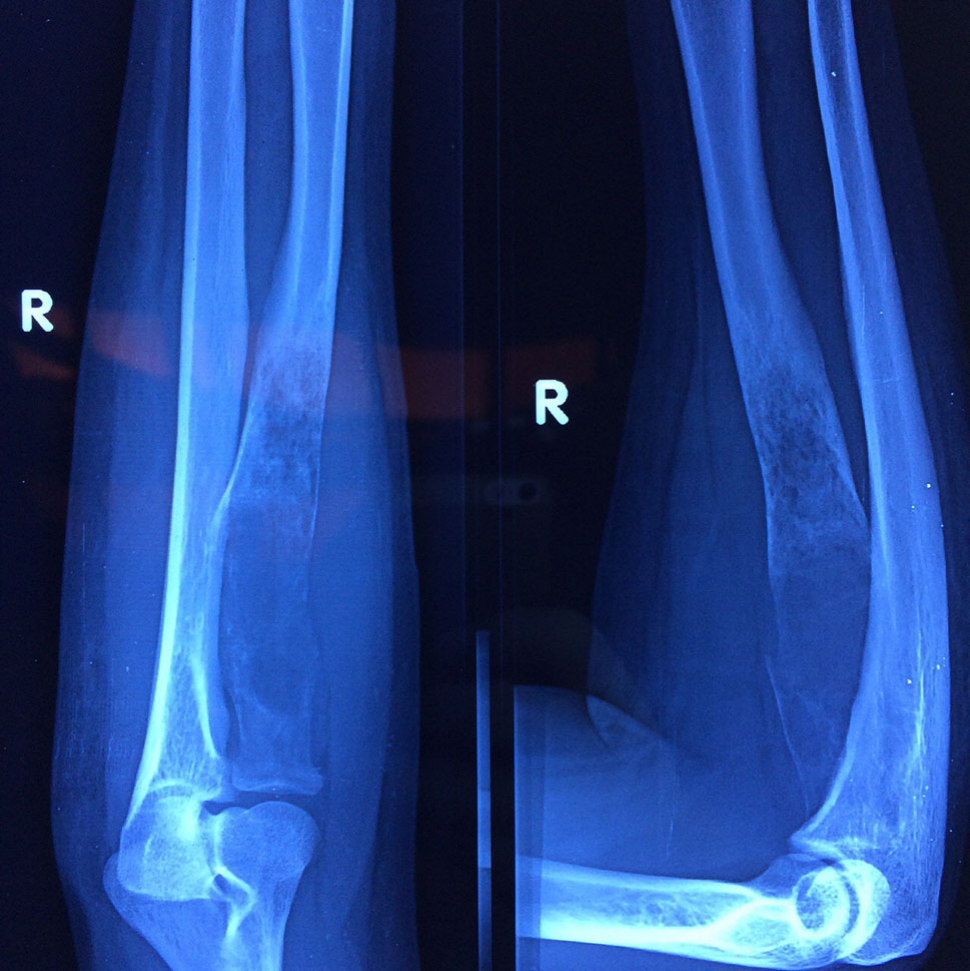
X-ray of right elbow with lytic lesion in the proximal radius

**Fig. 2:**
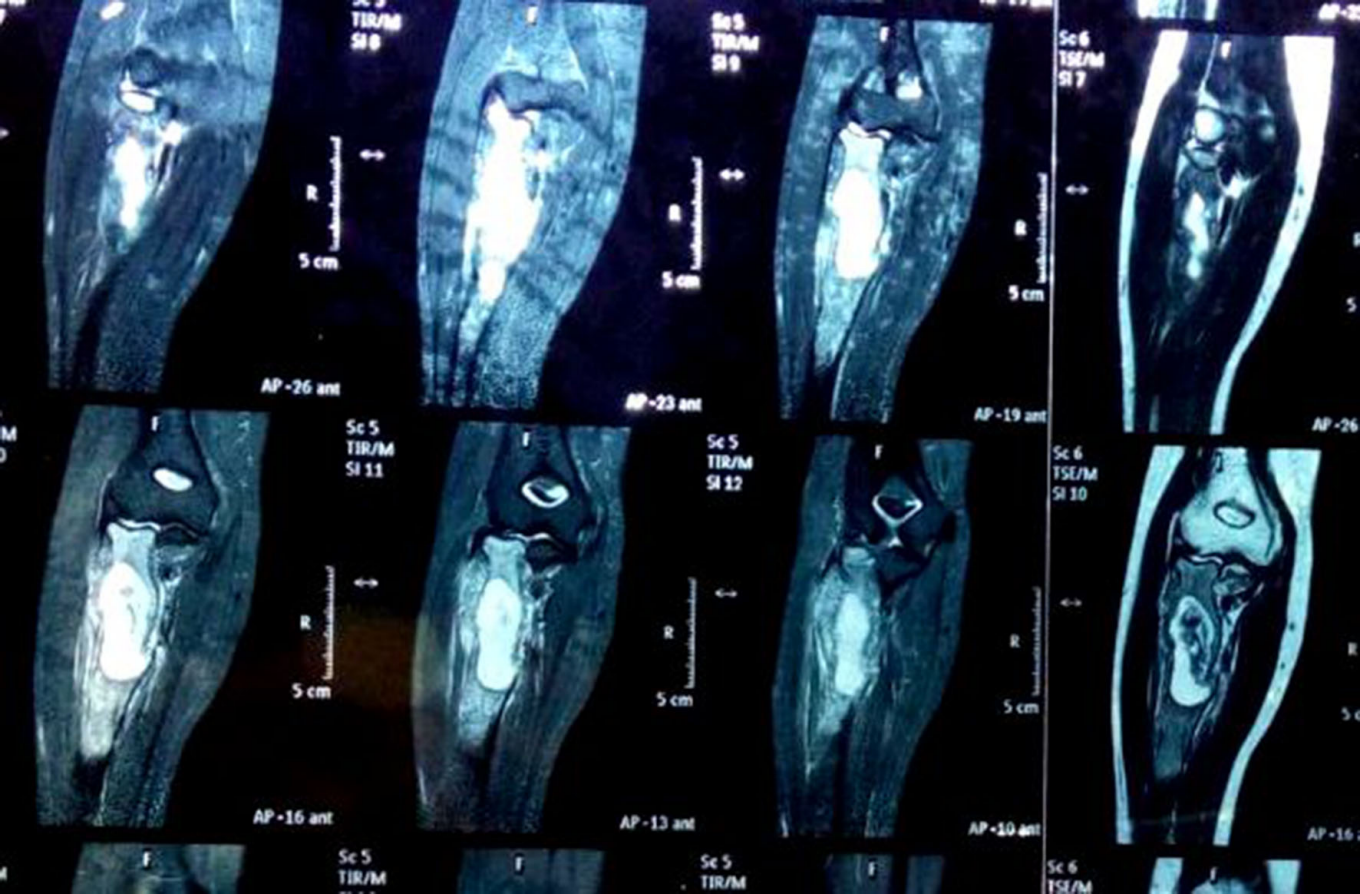
MRI sagittal view with hyperintense areas with lytic areas extending extraosseous

**Fig. 3:**
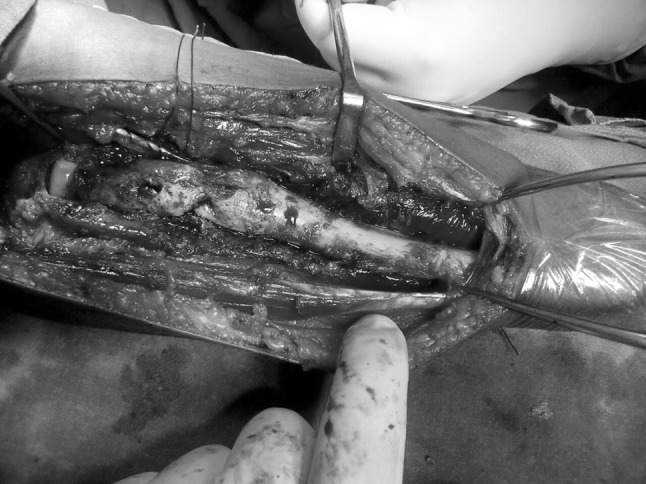
Intraoperative picture of pathological radius with lytic and necrotic areas

A 50-year-old female presented to our department with a swelling over the anterolateral aspect of right elbow along with wrist drop in February 2014. Roentgenogram was taken which showed expansile lesion in the metaphyseal region of proximal radius with the rim of cartilage and ballooning of proximal radius (Fig. 1). It was radiologically diagnosed as giant-cell tumour. Her MRI of forearm was performed to plan the surgery (Fig. 2). Needle biopsy on cytology confirmed the diagnosis. She was then counselled for wide margin resection and informed consent was taken. Elective surgery was performed in April 2014 which showed lytic and necrotic bone (Fig. 3). Wide margin excision was done with non-vascularised fibular graft reconstruction along with TENS (titanium elastic nail system). Cancellous bone graft taken from iliac crest was placed at the ends of fibular graft. Posterior interosseous nerve decompressed while removing the tumour. Annular ligament repaired and biceps tendon was passed through tunnel in fibular graft proximally and reattached with ethibond sutures stabilising the proximal radioulnar joint.

**Fig. 4:**
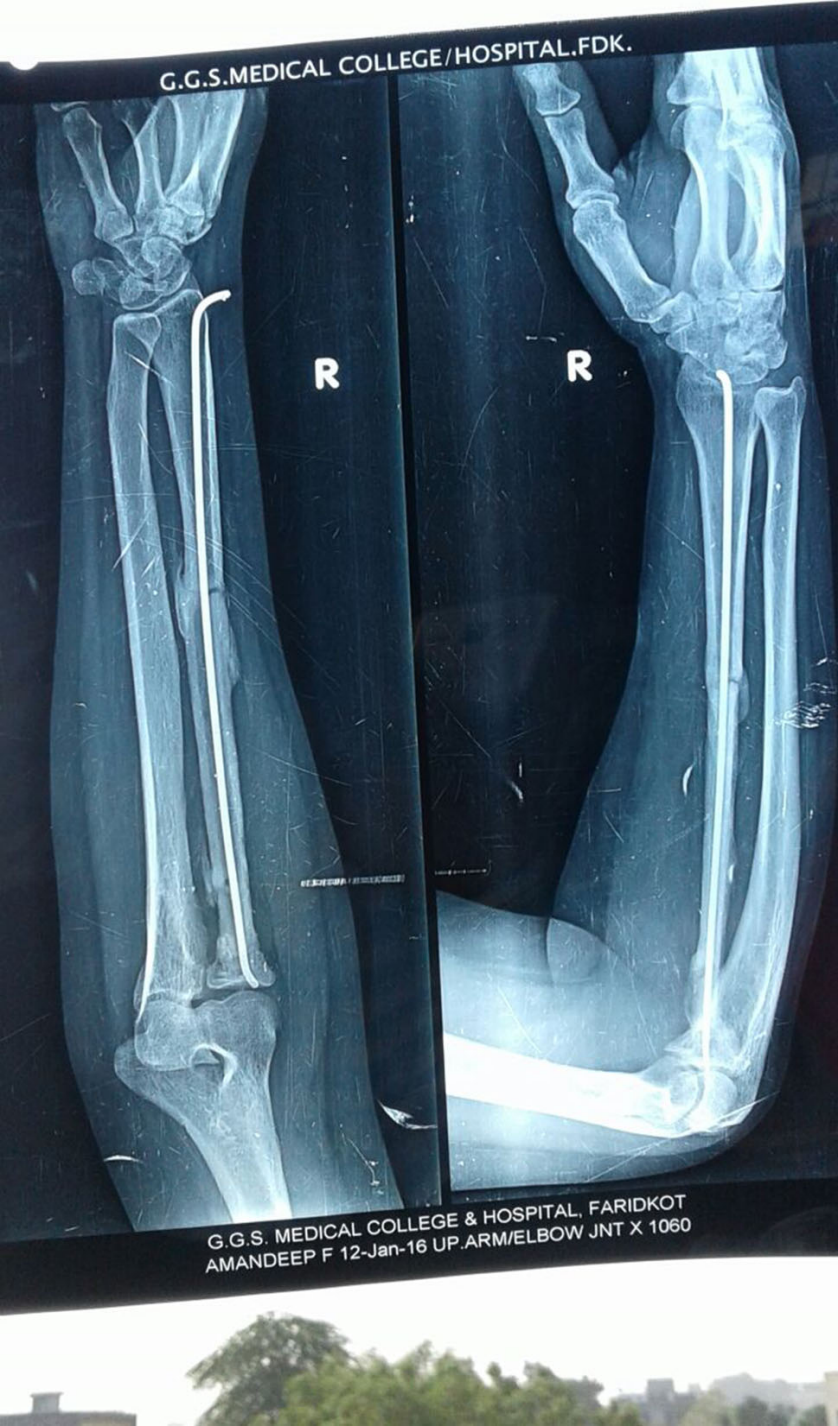
One-and-half-year-old post-operative X-ray with fibular graft along with TENS

Proximal interosseous membrane left untouched as distal diaphyseal interosseous membrane was intact and radioulnar joint was stable enough in supination and pronation. She did not have wound problems post-operatively but had weakness in finger and wrist extension for which cock-up splint and physiotherapy was advised. She has been under regular clinical and radiological follow-up. After 2 months of follow-up, she regained her finger and wrist movements. Bone scan conducted 9 months after surgery did not show any recurrence. There are no clinical or radiological symptoms (as shown in Fig. 4) and signs of recurrence of tumour till her last follow-up visit in January 2016. Elbow was stable in all range of motion, and she did not have any problem in daily routine activities.

## Discussion

Giant-cell tumour is mostly found in the third and fourth decade of life though it has been rarely seen in younger age group also. It is locally aggressive tumour involving epiphyseal region of mature bones. Most of the tumours are found around knee joint in distal femur, proximal tibia, and distal radius. Various treatment options are used depending on the stage and location of tumour. Four cases have been reported so far in the literature which shows unusual age and presentation in proximal radius. Akmaz et al., Mir, Singh, and Song [6–9] have reported giant-cell tumour of proximal radius and its management. Age of presentation in our case and that reported by Singh was almost the same and outside usual age range while other patients were from usual age group [8].

In our patient, the giant-cell tumour was extracompartmental invading surrounding muscle fibres and stretching posterior interosseous nerve, which had to be removed. Cases reported by above-mentioned authors were also extracompartmental tumours. Mir did marginal resection, and Singh performed above-elbow amputation [7, 8]. Song [9] did en bloc resection with reconstruction of proximal radius with polyethylene insert, screw, pins, and bone cement. Akmaz et al. [6] treated the intraosseous tumour in their case by curettage and bone grafting.

Dell et al. [10] and Brown [11] found no substantial difference between non-vascularised and vascularised grafts as far as consolidation duration or incidence of union is considered. Vascularised grafts were transiently stronger than conventional grafts in the first 6 months, but there was no difference thereafter. The complication rate for vascularised grafts has been reported to vary between 7 and 35% [12]. It appears to be higher than for non-vascularised grafts whose complication rate has been reported to vary between 4 and 12%. So we preferred non-vascularised free fibular graft.

 Gokaraju et al. [13] found good midterm results in a case series (five patients of proximal radius tumours) with metal proximal radial endoprosthesis instead of fibular grafting and found good post-operative stability of elbow and functional score (mayo elbow performance score of 86% considered as good).

According to Izaak et al. [14], the main limitation with current radial head prosthesis (RHP) designs is that only short- to midterm results are known. RHP may be classified according to the different materials used, and they are as follows: (silicone, polyethylene, pyrocarbon, metal), differences in modularity (monoblock vs. modular), polarity (uni- or monopolar vs. bipolar) or fixation method (cemented vs. uncemented press fit vs. intentional loose fit). Despite the growing amount of data, evolving surgical technique, and improving implant design and rationale, prosthetic radial head replacement is far from what should be considered an established and routine procedure. Regarding radial head prosthesis, cost factor is a big issue especially in developing countries. So we did not keep this as an option.

In our patient on last follow-up, she had nearly complete range of flexion/extension movements at elbow, supination/pronation, wrist, and fingers after extensive physiotherapy. Spared radial head showed no signs of avascular necrosis with good radiological union. Repair of annular ligament and biceps tendon reattachment on proximal fibula along with intact interosseous membrane of the distal diaphysis contributed to the desired stability of proximal radioulnar joint which is vital for supination and pronation movements and plays a role in valgus stability. There were no signs of recurrence. She was satisfied with her treatment. Early detection, wide margin resection of extracompartmental tumour along with fibular, and iliac bone grafting with TENS are good options for giant-cell tumour of proximal radius with no recurrence, minimal complications, and disability.
